# Targeted Delivery of Chemotherapeutic Agents for Osteosarcoma Treatment

**DOI:** 10.3389/fonc.2022.843345

**Published:** 2022-03-04

**Authors:** Duoli Xie, Zhuqian Wang, Jie Li, De-an Guo, Aiping Lu, Chao Liang

**Affiliations:** ^1^ Department of Biology, School of Life Sciences, Southern University of Science and Technology, Shenzhen, China; ^2^ Institute of Integrated Bioinfomedicine and Translational Science (IBTS), School of Chinese Medicine, Hong Kong Baptist University, Hong Kong, Hong Kong SAR, China; ^3^ Law Sau Fai Institute for Advancing Translational Medicine in Bone and Joint Diseases, School of Chinese Medicine, Hong Kong Baptist University, Hong Kong, Hong Kong SAR, China; ^4^ Department of Laboratory Medicine, Peking University Shenzhen Hospital, Shenzhen, China; ^5^ National Engineering Laboratory for Standardization of Traditional Chinese Medicine, Shanghai Institute of Materia Medica of the Chinese Academy of Sciences, Shanghai, China; ^6^ Institute of Arthritis Research in Integrative Medicine, Shanghai Academy of Traditional Chinese Medicine, Shanghai, China; ^7^ Guangdong-Hong Kong-Macau Joint Lab on Chinese Medicine and Immune Disease Research, Guangzhou, China

**Keywords:** osteosarcoma, targeted delivery, chemotherapeutic agents, ligand-based delivery systems, antibodies

## Abstract

Since osteosarcoma (OS) is an aggressive bone cancer with unknown molecular pathways of etiology and pathophysiology, improving patient survival has long been a challenge. The conventional therapy is a complex multidisciplinary management that include radiotherapy, chemotherapy which followed by surgery and then post-operative adjuvant chemotherapy. However, they have severe side effects because the majority of the medicines used have just a minor selectivity for malignant tissue. As a result, treating tumor cells specifically without damaging healthy tissue is currently a primary goal in OS therapy. The coupling of chemotherapeutic drugs with targeting ligands is a unique therapy method for OS that, by active targeting, can overcome the aforementioned hurdles. This review focuses on advances in ligands and chemotherapeutic agents employed in targeted delivery to improve the capacity of active targeting and provide some insight into future therapeutic research for OS.

## Introduction

**Figure 1:**
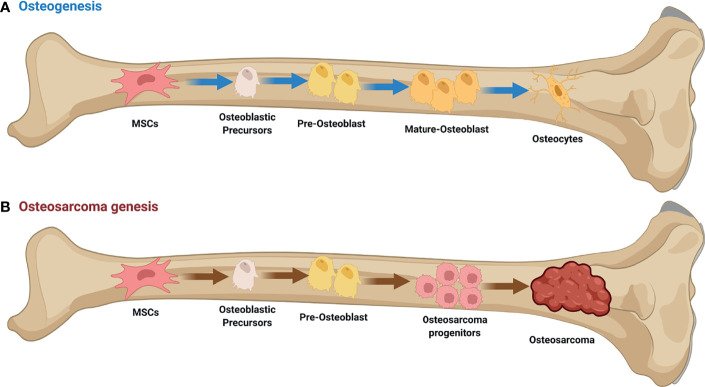
Osteogenesis and Osteosarcoma genesis. **(A)** Initiation of osteogenic differentiation from mesenchymal stem cells (MSCs). MSCs are multipotent bone marrow cells that are capable of differentiating to bone (osteoblast/osteocyte), fat (adipocyte), and cartilage (chondrocyte) tissues. **(B)** Defects in osteogenesis lead to osteosarcoma genesis. Genetic alterations probably interfere with the normal osteogenic process, resulting in incompletely differentiated osteoblasts or osteocytes in bone. These defects disrupt the balance between proliferation and differentiation, and may cause a group of cells to display uncontrolled cell proliferation. Osteosarcoma progenitors may arise from these cells and expand to form osteosarcoma. This figure was created with BioRender.com.

Osteosarcoma (OS) is a relatively rare malignant mesenchymal origin bone cancer that 70%–80% of OS patients are adolescents and young adults and is distinguished by the formation of immature osteoid extracellular matrix (1). The disease has a 1–3 case per 1,000,000 population incidence and accounts for 20% of all primary malignant bone tumors in the world (2). OS is most common in the metaphysis of long, tubular bones like the proximal humerus, distal femur, and proximal tibia. It is very rare in the spine, pelvis, and sacrum, but it can be found in the metaphysis of some other bones (3). More than 85% of individuals with localized illness will develop a local or distant recurrence, most commonly in the lungs (85-90%) (4). Clinically, the development of disease is marked primarily by local discomfort and swelling, with occasional joint dysfunction (5) (**Figure 1**). 

Chemotherapy, followed by total surgical resection and then post-operative adjuvant chemotherapy as well as radiotherapy, is currently the standard treatment strategy for OS. Methotrexate (MTX), doxorubicin (DOX), cisplatin (CDDP), ifosfamide (IFO), and etoposide are commonly used chemotherapeutic agents recommended by The National Comprehensive Cancer Network guidelines, which increased the survival rate in patients with localized resectable tumors by up to 60-70% (1); However, most clinical applications of chemotherapeutics for patients with advanced and metastatic OS have been limited due to a lack of selectivity and sensitivity to tumor cells, toxicity towards normal cells, multidrug resistance (MDR), poor pharmacokinetic performance (6), and other factors that limit treatment efficacy and result in severe adverse effects on vital organs (7). With these combination therapy, the overall 5-year survival percentage for individuals with primary metastatic or relapsed cancer is less than 20% (8). Despite numerous clinical trials over the last three decades, cure rates for those with OS have not considerably improved, and survival for patients with metastatic or recurrent disease remains bleak (9). In the meantime, there are many NCT clinical trials in progress, pending follow-up (**Table 1**)

**Table 1:** Ongoing NCT clinical trials of OS.

Clinical Trial NCT No.	Phase	Title	No. of patients	Status; Estimated completion date	Cancer Type	Sponsor
NCT01459484	II	ABCB1/P-glycoprotein Expression as Biologic Stratification Factor for Patients with non metastatic Osteosarcoma (ISG/OS-2)	225	Active, not recruiting; October 30, 2021	Non-metastatic extremity high-grade osteosarcoma	Italian Sarcoma Group
NCT03006848	II	A Phase II Trial of Avelumab in Patients with Recurrent or Progressive Osteosarcoma	19	Active, not recruiting; January 31, 2023	Recurrent/Refractory osteosarcoma	St. Jude Children’s Research Hospital
NCT04154189	II	A Study to Compare the Efficacy and Safety of Ifosfamide and Etoposide with or Without Lenvatinib in Children, Adolescents and Young Adults with Relapsed and Refractory Osteosarcoma	72	Active, not recruiting; December 31, 2022	Relapsed or Refractory Osteosarcoma.	Eisai Inc.
NCT02484443	II	Dinutuximab in Combination with Sargramostim in Treating Patients with Recurrent Osteosarcoma	41	Active, not recruiting; N.A.	Metastatic Malignant Neoplasm in the Lung Metastatic/Recurrent Osteosarcoma	National Cancer Institute (NCI)
NCT02470091	II	Denosumab in Treating Patients with Recurrent or Refractory Osteosarcoma	56	Active, not recruiting; September 30, 2022	Metastatic/Recurrent/Refractory Osteosarcoma Stage IV/IVA/IVB Osteosarcoma AJCC v7	Children’s Oncology Group
NCT02432274	I/II	Study of Lenvatinib in Children and Adolescents with Refractory or Relapsed Solid Malignancies and Young Adults with Osteosarcoma	117	Active, not recruiting; March 31, 2022	Tumors Solid Malignant Tumors Osteosarcoma Differentiated Thyroid Cancer (DTC)	Eisai Limited
NCT02243605	II	Cabozantinib S-malate in Treating Patients with Relapsed Osteosarcoma or Ewing Sarcoma	90	Active, not recruiting; N.A.	Metastatic/Recurrent/Unresectable Ewing Sarcoma Recurrent/Metastatic/Unresectable Osteosarcoma Stage III/Stage IV/Stage IVA/Stage IVB Osteosarcoma AJCC v7	National Cancer Institute (NCI)
NCT04690231	N.A.	Apatinib + Ifosfamide and Etoposide for Relapsed or Refractory Osteosarcoma	79	Active, not recruiting; June 1, 2021	Relapsed or Refractory Osteosarcoma	Peking University People’s Hospital
NCT00470223	III	Combined Chemotherapy With or Without Zoledronic Acid for Patients With Osteosarcoma (OS2006)	318	Active, not recruiting; December 2025	Osteosarcoma	UNICANCER
NCT01953900	I	iC9-GD2-CAR-VZV-CTLs/Refractory or Metastatic GD2-positive Sarcoma and Neuroblastoma (VEGAS)	26	Active, not recruiting; October 31, 2034	Osteosarcoma Neuroblastoma	Baylor College of Medicine
NCT02357810	II	Pazopanib Hydrochloride and Topotecan Hydrochloride in Treating Patients With Metastatic Soft Tissue and Bone Sarcomas	178	Active, not recruiting; June 2022	Adult/Metastatic/Recurrent Liposarcoma Metastatic/Recurrent Osteosarcoma Recurrent /Stage IV Adult Soft Tissue Sarcoma	Northwestern University

As a result, the development of some new anticancer medicines with reduced toxicity and higher tumor-killing effectiveness is desirable in order to increase patient survival and quality of life (10). A potent treatment with rational delivery vehicle and a surface ligand are often included in a ligand-based drug delivery system (11). Drugs might be delivered to tumor sites by receptor-mediated endocytosis once the individual ligands have contacted the corresponding tissue *in vivo*, allowing for tailored distribution of unique effector molecules while reducing adverse effects (12). Ligands such as antibodies, aptamers, peptides (13), saccharide, vitamin, bisphosphonates (BP) (14, 15), hyaluronic acid (HA) (16) and folate (17) have been reported to be used in the development of OS targeted drug delivery systems that mediate delivery vehicle and drug interactions with and internalization into OS cells with high specificity and efficiency (7). Some of them have a strong affinity for hydroxyapatite and can be employed as ligand in bone (7). In terms of drug carrier, liposomes which Alec D. Bangham developed in 1965, served as the first therapeutic nanoparticles to receive FDA approval, are now the most widely used (18). Liposomes have been extensively explored since they are capable of holding both hydrophilic and lipophilic pharmaceuticals (19). Mepact–liposomal mifamurtide which had been commercialized by Takeda Pharmaceuticals in 2004, is indicated for the treatment of high-grade, nonmetastasizing, resectable osteosarcoma following complete surgical removal in children, adolescents, and young adults (20). For further development in the similar line of thought, liposomes combined with chitooligosaccharides with a disulphide linker were developed by Yin et al. (21); Similarly, a reduction-responsive liposome decorated with COS and functionalised with oestrogen has been synthesised to preferentially target MG63 cells (22); Haghiralsadat et al. synthesised a thermo- and pH- sensitive liposome for nanoformulation of DOX which could inhibit the proliferation of MG63 cells and reduce cytotoxicity to healthy bone cells (23). As above mentioned, the advantages of traditional anti-OS chemotherapy are expected to be overcome by these drug delivery systems. In this review, we will look at the different types of ligands that can specifically bind to the matching receptors in OS and cause receptor-mediated endocytosis. Furthermore, the drug conjugates produced by chemically conjugating drugs were explained using studies that were available. This will spark new ideas for the development of more effective therapeutic options.

## Main Content

### Antibodies as Targeting Ligands

Antibody-drug conjugates (ADCs) are monoclonal antibodies (mAbs) that have been chemically linked to cytotoxic medications and are directed onto a cancer cell surface antigen, delivering and releasing cytotoxic chemicals at the tumor site with low systemic toxicity (24, 25). The FDA has currently approved a number of ADCs for clinical use in cancer therapy (26). In addition, the ADCs are well equipped for pharmacological agents in OS.

Microtubules are dynamic filamentous cytoskeletal proteins. For decades, until the advent of targeted therapy, microtubules were the only alternative to DNA as a therapeutic target in cancer (27). The most widely used pharmacological agents conjugated to antibodies are DNA-targeting and tubulin-targeting medicines, which cause DNA alkylation or double-strand break and prevent tubulin depolymerization, respectively (28).

The cytotoxic component of the dimeric protein ricin, one of the most potent and deadliest plant poisons generated from Ricinus communis seeds, is A chain (RTA). RTA has been investigated as a potential option for cancer treatment in the form of immunotoxins, as well as an approach for *in vivo* macrophage depletion (29). Gp72 is a cell surface antigen discovered on the surface of tumors such as osteogenic sarcomas (30). The anti-gp72 mAb 79IT/36 conjugates with MTX or RTA as therapeutic moieties demonstrate substantial results in treating OS (30), and better results were obtained with 79IT/36-RTA. Mice treated with the 79IT/36-RTA immunotoxin for five days, tumor growth was significantly suppressed *in vivo*.MM

The transmembrane protein glycoprotein non-metastatic b (gpNMB) plays a physiological role in bone differentiation and remodeling. gpNMB was found to be abundant in OS samples, implying that gpNMB could be a suitable target for antibody-mediated drug delivery in OS (31). Glembatumumab vedotin is an ADC that combines the anti-gpNMB mAb targeting characteristics with the payload of the antimitotic microtube inhibitor MMAE. The glembatumumab vedotin has shown strong preclinical activity in *in vitro* and *in vivo* studies (32), whereas in a phase II clinical trial, it shows limited efficacy in relapsed and/or refractory OS (31).

CD184 (CXCR4) is a G-protein coupled receptor discovered on the surface of metastatic tumor cells. In patients, CXCR4 expression is a poor predictor of survival and a high predictor of tumor relapse (33). It could be efficiently absorbed when the ligand binds. A recombinant anti-CD184 mAb coupled to MMAE demonstrated significant toxicity *in vitro* on metastatic OS cells derived from lung metastasis (34). Similarly, an anti-CXCR4 IgG–auristatin ADC demonstrated superior activity against metastatic SJSA-1-met-luc cells (OS lung metastasized cells from mouse) (35). This ADC is capable of eradicating tumors in mice receiving the immunoconjugate in a tumor xenograft lung-seeding model.

The CD248 (endosialin/TEM1) receptor is a transmembrane glycoprotein found on pericytes and fibroblasts during embryogenesis. It has been associated to tumor angiogenesis and inflammation, making it a molecular and therapeutic target for OS (36, 37). Two anti-endosialin ADCs were investigated in preclinical OS models (28). The anti-endosialin-MC-VC-PABC-MMAE was investigated for antitumor effectiveness in two endosialin-positive human cell lines and one sarcoma xenograft model. *In vitro*, a completely human anti-CD248 mAb coupled to MMAE inhibited the development of CD248 overexpressing OS cells (37).

TGF induces leucine-rich repeat containing 15 (LRRC15), a member of the Leucine-Rich Repeat superfamily, on activated fibroblasts (SMA+) and mesenchymal stem cells (MSC), which is associated with cell adhesion, invasion, and immunological responses. LRRC15 is a new cancer-associated fibroblast and mesenchymal marker that is overexpressed in OS tissue samples and is being investigated as a potential therapeutic target for ADC-based sarcoma treatment (38, 39). Samrotamab vedotin (ABBV-085) is an ADC made up of an anti-LRRC15 humanized IgG1 antibody (Ab1) linked to the anti-mitotic medication MMAE *via* a protease cleavable valine-citrulline (vc) linker. In preclinical studies, samrotamab vedotin extended event-free life in patient-derived xenograft (PDX) models (40), which may target cancer cells over LRRC15-positive cancer-associated fibroblasts due to the cell-permeable characteristics of MMAE. However, data from this unique stromal-targeting ADC’s phase 1 clinical trial are mixed (38).

CD13, also known as aminopeptidase-N (APN) and alanyl aminopeptidase (ANPEP), is a metallopeptidase that was initially discovered to be a myeloid-specific hematological marker (41). A number of studies have found that CD13 plays a role in tumor growth, metastasis, and angiogenesis. By conjugating anti-CD13 monoclonal antibody (mAb) TEA1/8 to the marine chemical PM050489, a novel ADC, MI130110, was created. The MI130004 shown exceptional effectiveness in numerous murine xenograft models for OS (42).

Endothelial growth factor (VEGF) antibody was chosen as the targeted agent for the elevated production of VEGF antigen in OS cells (43), which plays a significant role in tumor angiogenesis processes. For the treatment of OS, an iron oxide nanoparticle complex conjugated to VEGF antibody and the ligand cluster of differentiation 80 (CD80) was developed (44). This combination approach would be able to target not just the extensively expressed VEGF antigen in OS cells, but also the increased expression of the surface cell receptor cytotoxic T lymphocyte-associated antigen-4 (CTLA4) (44).

The anti-CD11c mAb interacted with the CD11c receptor, which is abundant in OS cell lines. The functionalized nanoparticles of mesoporous silica nanoparticles (MSNPs) loaded with DOX and coupled with the anti-CD 11c mAb may considerably boost cellular absorption, resulting in an increased toxic and antiproliferative potential (45).

A transmembrane glycoprotein from the immunoglobulin family, activated leukocyte cell adhesion molecule (CD166/ALCAM), can be employed as a cell surface receptor for targeting OS. In SCID mouse xenograft models, the anticancer efficacy of a mAb anti-CD166 conjugation to DOX-loaded liposomal nanoparticles targeting CD166 in OS cell lines was investigated *in vivo*. When compared to non-targeted medicines, these antibody-targeted medications demonstrated an increase in cytotoxicity for OS cells (7, 13).

**Figure 2:**
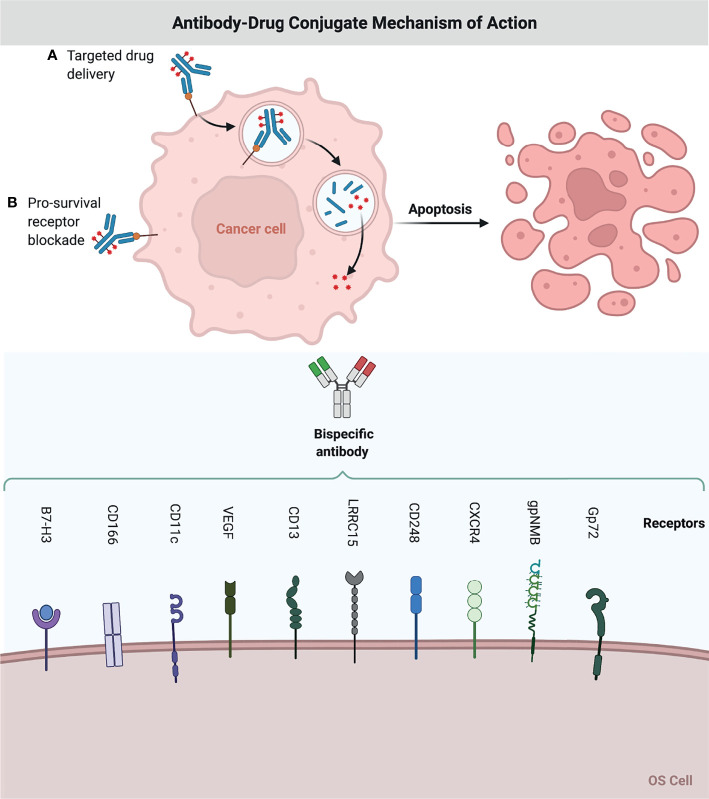
Antibody-Drug Conjugate Mechanism of Action. A chemodrug is coupled to an antibody that specifically targets a certain OS antigen. Antibodies attach themselves to the antigens on the surface of cancerous cells. The biochemical reaction between the antibody and the target protein (antigen) triggers a signal in the OS cell, which then inhibits OS cell growth, or internalizes the antibody together with the linked chemo drug and eliminates the OS cell. This figure was created with BioRender.com.

The B7-H3 receptor was significantly overexpressed in OS specimens, implying the possibility of targeting this receptor for therapeutic purposes (46). ADCs targeting B7-H3 are also being developed, such m276-PBD (47). This drug carries a PBD payload containing a DNA-damaging agent and elicited full responses in two of five OS PDX models (47). MGC018 is another ADC targeting B7-H3 that has a DNA alkylating payload (duocarmycin) that is now being tested in OS PDX models (48). Preliminary data showed a controllable safety profile and signs of action in four of the twenty OS patients included. (**Table 2** and **Figure 2**)

**Table 2:** Targeted delivery based on antibody as ligands.

Ligands	Targets	Therapeutic agents	References
gp72 mAb	gp72	MTX/RTA	(30, 49)
gpNMB mAb	gpPNMB	MMAE	(32)
anti-CD184 mAb	CXCR4	MMAE/auristatin	(35, 50)
anti-endosialin Ab	CD248	MMAE	(28, 37)
anti-LRRC15 humanized IgG1 kappa antibody Ab1	LRRC15	MMAE	(38, 40)
anti-CD13 mAb	CD13	PM050489	(42)
anti-VEGF mAb	VEGF	N/A	(44)
anti-CD11c mAb	CD11c	DOX	(45)
anti-CD166 mAb	CD166	DOX	(7, 13)
B7-H3 mAb	CD276	PBD/duocarmycin	(47, 48)

gpNMB, glycoprotein non-metastatic b; CD, cluster of differentiation; LRRC15, Leucine-rich repeat containing 15; VEGF, Vascular endothelial growth factor; MTX, Methotrexate; RTA, ricin toxin A chain; ﻿MMAE, monomethyl auristatin E; DOX, Doxorubicin; PBD, pyrrolobenzodiazepine.

### Aptamers as Targeting Ligands

Aptamers are single-stranded, synthetic DNA or RNA molecules that may fold into unique three-dimensional conformations to attach to specific target molecules with great affinity (51). Through SELEX, a repetitive *in vitro* process of sequential selection and amplification steps, aptamers could be selected from DNA or RNA libraries and function like “chemical antibodies”. They are excellent candidates for targeted delivery of therapeutic agents due to their high selectivity and specificity, low immunogenicity, ease of synthesis with low cost and high reproducibility, relatively rapid tissue penetration with no toxicity (52, 53); As a result, they are typically utilized in conjunction with anticancer medications to target tumor cell surfaces (7), which may result in more promising outcomes when compared to aptamer-free competitors. Aptamer-drug conjugates (ApDCs) are an efficient technique of reducing OS growth *in vitro* and *in vivo* due to advances in technology (54).

Vascular Endothelial Development Factor A (VEGF A) overexpression was linked to tumor growth and angiogenesis. Our research has created an OS cell-targeted aptamer (LC09) that binds to VEGFA-positive K7M2 OS cells but not to mice normal hepatocytes (AML12) or peripheral blood mononuclear cells (PBMCs). After *in vivo* delivery, this aptamer may reduce non-specific liver and PBMC uptake. LC09-modified lipopolymers that were loaded with CRISPR/Cas9 plasmids containing VEGFA gRNA and Cas9 showed that they were only found in OS and lung metastasis. This led to a decrease in VEGFA expression and secretion, as well as a decrease in OS malignancy and lung metastasis (55, 56).

CD133 is a transmembrane glycoprotein that is thought to be a cancer stem cell (CSC) marker in OS and other cancers (57–59). As a result, CD133 aptamers have been employed as targeted ligands for OS CSC monitoring (60, 61). CD133-functionalized polymeric nanoparticles loaded with salinomycin could precisely and efficiently transport anticancer medicines to CD133 positive OS CSCs, greatly inhibiting OS development by eliminating CD133+ OS CSCs (62).

**Figure 3:**
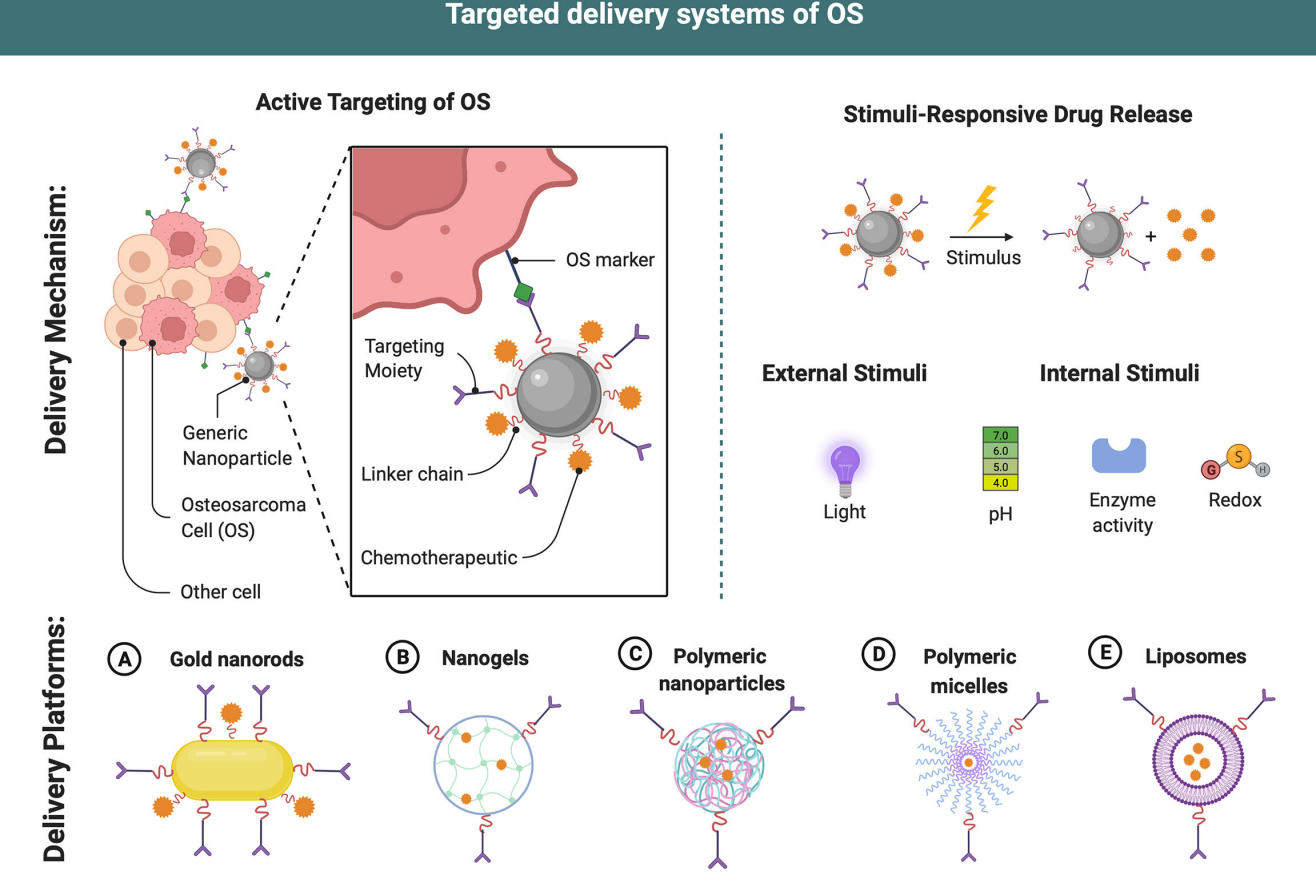
Targeted delivery systems of OS. Different delivery systems have ideal properties for chemodrugs transport and delivery. Targeting ligands may be attached to the surface allowing an active targeting strategy and an increase in efficiency of the therapeutic payloads in OS therapy. After internalization, dissociation occurs at a proper microenvironment due to different stimuli (enzyme, redox, etc.), drug payloads are released into the cytosol of cancer cells. This figure was created with BioRender.com.

Due to the fact that amplification of the epidermal growth factor receptor (EGFR) is a frequent genetic aberration in OS, EGFR became a feasible target in the disease. EGFR aptamers were used to create OS-targeted medication delivery vehicles. Yu et al. (63) found that EGFR-SNPs, which are aptamer-conjugated polymer-lipid hybrid NPs that are loaded with salinomycin, effectively stopped the formation of tumorspheres and reduced the number of CD133-positive OS CSCs. This led to an even stronger cytotoxic effect than with non-targeted SNPs and salinomycin. Chen et al. (60) engineered salinomycin-entrapped lipid-polymer nanoparticles (CESP) with CD133 and EGFR aptamers to specifically target OS cells and CSCs. CESP demonstrated superior cytotoxicity to single-targeted or untargeted salinomycin-loaded nanoparticles in OS cells and CSCs. In OS-bearing mice, *in vivo* administration of CESP inhibited tumor growth more than other controls (**Table 3** and **Figure 3**).

**Table 3:** Targeted delivery based on aptamers, peptides, saccharide, vitamin or bisphosphonates as ligands.

Ligands	Targets	Therapeutic agents	References
LC09 aptamer	VEGFA	plasmids encoding VEGFA gRNA and Cas9	(56)
CD133 aptamer	CD133	salinomycin	(60–62)
EGFR aptamer	EGFR	salinomycin	(60, 63)
YSA peptide	EphA2	DOX	(64)
VIP peptide	VPAC1R and VPAC2R	DOX	(65)
RGD peptide	Integrins	DOX	(13, 66, 67)
iRGD peptide	NRP-1	N/A	(68, 69)
KRP	RPS6KA2	DOX	(70, 71)
HA	CD44	DOX	(16, 16, 72)
FA	FRs	MTX and/or DOX	(73–76)
Alendronate	/	DOX or PTX	(77–79)
Bisphosphonate prodrug	/	DOX	(80)
Phospholipid	/	MDP	(81)
Bisphosphonate	/	DOX	(56, 82)
Pamidronate	hydroxyapatite	DOX	(83)
Medronate	/	DOX	(77, 82)
pamidronate	/	DOX	(84)

VEGFA, Vascular Endothelial Growth Factor A; EGFR, epidermal growth factor receptor; EphA2, ephrin type-A receptor 2; VIP; vasoactive intestinal peptide; RGD, Arg-Gly-Asp; CD, cluster of differentiation, DOX; doxorubicin; iRGD, Internalizing Arg-Gly-Asp; NRP-1, αv integrins and neuropilin-1; HA, Hyaluronic acid; FA, Folate or folic acid; FRs, folate receptors; MTX, methotrexate; MDP, Methylene diphosphonate.

### Peptides as Targeting Ligands

Peptides are short chains of amino acids linked by peptide bonds that are typically thought to be harmless due to their low immunogenicity and non-toxic metabolites (85). Peptides are thought to be swiftly broken by proteolytic enzymes and removed from the bloodstream by the liver and kidney. Different ways to modification and stabilization can be used to alter these pharmacodynamic features (86). Lipidation, which involves the incorporation of fatty acids into the peptide, is one of the most well-known principles in peptide stabilization. Fatty acids attachment could induce a more extended circulation period (87). As carrier molecules, peptide-drug conjugates (PDCs) have various advantages. The straightforward synthesis allows for reasonable optimization of side chains and backbone structures, which might result in improved binding affinities and direct influence of physicochemical attributes (88). Furthermore, their low molecular weight allows for greater penetration into solid tissues, leading in a more effective anti-tumor impact (89, 90).

Conventional chemotherapeutic medicines linked with peptides present greater pharmacokinetics and reduced cytotoxic. Chemotherapeutic drugs utilized in PDCs are divided into three types. The chemicals first bind to and interact with cellular DNA or DNA-protein complexes. As a result, transcription and DNA replication are disrupted, resulting in the activation of apoptosis. The cytotoxicity of the second class of commonly used toxophores is conveyed through blocking DNA biosynthesis. These antimetabolites include the folate derivative MTX, which inhibits the dihydrofolate reductase enzyme. Anti-mitotic drugs, which operate on microtubules, comprise the third class of chemotherapeutics (91).

By loading the same medication DOX, several new peptide-based targeted delivery systems were produced. The 12-amino acid peptide YSA (YSAYPDSVPMMS) is an ephrin A1 mimic and ligand for ephrin type-A receptor 2. (EphA2). Ephrins are a large family of tyrosine kinase receptors with well-documented roles in cancer proliferation and metastasis. They are emerging as intriguing prospective targets for cancer therapeutic methods (92, 93). Surface molecule EphA2a is extensively expressed in both primary and metastatic OS cells (94). In comparison to doxorubicin-free and non-targeted L-doxorubicin, DOX-loaded liposomes modified with the YSA peptide may more effectively target human Saos2 OS cells, hence increasing toxicity and cellular absorption (64). When D-aspartic acid octapeptide conjugate to micelles includes the medication DOX, it effectively promotes DOX accumulation in OS while having minimal side effects (65). The vasoactive intestinal peptide (VIP) receptors (VPAC1R and VPAC2R) are significantly expressed in Saos2. To treat OS cells, a new lipid analog (lipopeptide) coupled with VIP was created. It was proposed that the lipopeptide could be a good molecule for OS (65).

RGD, an integrin selectivity and affinity tripeptide, can be employed as an effective cancer therapeutic ligands as it can accomplish dual targeting for angiogenic endothelial cells and various tumor cells *via* the receptors integrin v3 and v5, respectively (95, 96). Integrins, which are abundantly expressed in OS cell lines, connect the extracellular matrix to the intracellular cytoskeleton to mediate cell adhesion, migration, and proliferation (97). RGD peptides have the advantage of having a low risk of immunological reactivity, being simple and affordable to synthesize, and having tight control over ligand presentation (66), as well as active OS cell targeting capacity (13, 67). A study by Fang et al. (13) found that RGD-DOX polymeric micelles were more effective in eliminating osteoblasts than nontargeted micelles, demonstrating their ability to target and kill OS cells *in vitro*. Beyond RGD, internalizing Arg-Gly-Asp peptide (iRGD) combines RGD’s tumor-homing ability with C-end Rule’s tissue penetrating characteristic, allowing for the targeting of extravascular tumor parenchyma (68). The iRGD mechanism consists of three steps: The RGD motif binds to v integrins on tumor endothelial cells, and subsequently iRGD is proteolytically cleaved, gaining the ability to bind to neurophilin-1 and so achieving tissue penetration (68). Malignancies overexpressing v integrins and neuropilin-1 that internalize RGD (iRGD) may have increased vascular and tissue permeability (NRP-1). Increased expression of v integrins and NRP-1 in OS may serve as a predictor for therapeutic treatment optimization through the discovery of these two genes (69).

It was found that when KRP-DOX was combined with doxorubicin, it had multiple synergistic functions *in vitro* and *in vivo*, including good biocompatibility and biodistribution, selective accumulation of tumor tissues, and an ability to remain in tumor tissues and be internalized by cancer cells in the presence of KRP. KRP-DOX complex also evaded lysosomal breakdown and exhibited cytotoxicity in OS cells (70). OS mice were given KRP-DOX, which was shown to be more effective than either saline or DOX alone in controlling RPS6KA2 expression (71) (**Table 3** and **Figure 3**).

### Saccharide as Targeting Ligands

Proteoglycans, such as glycoproteins, are typically found on the outer surface of cancer cells, making saccharides or polysaccharides ideal ligands for OS-targeted drug delivery (98).

Biodegradable and biocompatible linear polysaccharides, such as hyaluronic acid (HA), are naturally biodegradable and biocompatible linear polysaccharides composed of glucuronic acid and N-acetyl d-glucosamine linked by alternating -1,4 and -1,3 glycosidic connections (99–102). The cluster determinant 44 (CD44) HA receptor is substantially expressed on MG-63 cells (103, 104), implying that HA could be a promising targeting agent for drug delivery in OS treatment. Nanocarriers with HA-CD44 interactions have recently been employed for tumor-targeted medicine delivery due to the obvious leaky vasculature of solid tumors (105, 106). Redox-sensitive, HA-functionalized liposomal nanocarriers have been developed by Chi et al. (16) to improve OS therapy. It was shown that HA-modified liposomes were more able to penetrate OS MG63 cells than regular human hepatocytes. Non-HA-coated nanoparticles, on the other hand, showed a decrease in tumor formation and an increase in tumor suppression. Dox administration in OS therapy can be improved by using a CDDP-crosslinked HA nanogel (CDDPHANG) (72). Redox-sensitive and CD44-targeted liposomes were developed in another study (Chol-SS-mPEG/HA-L). Liposomes loaded with DOX were coated with noncovalent HA, showing that the easily manufactured Chol-SS-mPEG/HA-L was demonstrated to be an efficient intracellular drug delivery system that may circulate for long periods of time and release GSH-triggered cytoplasmic drug (16). Beyond HA, recently a study demonstrated that chitooligosaccharides modified liposome loaded with DOX presented a good therapeutic effect in MG63 cell-bearing nude mice (21) (**Table 3** and **Figure 3**).

### Vitamin as Targeting Ligands

Vitamins are a group of chemical molecules and nutrients that are essential for the survival of all living cells. Rapid multiplication of tumor cells, in particular, need an excess of specific vitamins, such as folate and retinoic acid (RA), in order to support their rapid development. On the tumor cell surface, the receptors involved in vitamin absorption are consequently increased when compared to normal cells. As a result, these vitamin receptors are useful target substrates for tumor-targeted medication delivery.

Coenzymes that assist the transfer of one-carbon units from donor molecules into essential biosynthetic pathways such as methionine, purine, and pyrimidine biosynthesis require folic acid (FA), also known as water-soluble vitamin B9, vitamin M and vitamin Bc (107, 108). Furthermore, it is involved in the interconversion of serine and glycine, as well as histidine catabolism (108). The principal method of cellular internalization *via* high affinity folate receptors (FRs) is receptor-mediated endocytosis. For ligand-based targeted treatment, folate receptor-targeted drug delivery vehicles have been revealed to transport anticancer medications into cells *via* receptor-mediated endocytosis, making FA an ideal alternative (109, 110). Through receptor-mediated endocytosis, high-affinity folate receptors (FRs) are involved in the cellular uptake of these nutrients (73). As a result, folate-functionalized nanocarriers were employed in OS-targeted treatment (17, 74, 111). Through the interaction of FA-FRs, these nanosystems may preferentially aggregate in tumor masses and suppress tumor development.

Nanocrystalline apatite substrates coupled with FA and MTX were employed in the human SAOS-2 OS cell line (75). For OS treatment, Ai et al. (112) produced FA surface modified-titanium dioxide NPs (FA-TiNP) that displayed a superior anticancer impact compared to TiNP.

Folate-targeted gold nanorods (GNRs) are being developed as an OS treatment. To act as coating agents for GNRs, an amphiphilic polysaccharide-based graft-copolymer (INU-LA-PEG-FA) and an amino derivative of the, poly(N-2-hydroxyethyl)-D,L-aspartamide functionalized with folic acid (PHEA-EDA-FA) were produced. In tridimensional (3-D) OS models, the role of folate-targeted GNRs is investigated (111). Another study looked at the anticancer potential of curcumin and C6 ceramide (C6) when both were enclosed in a bilayer of liposomal nanoparticles. With C6-curcumin-FA liposomes, a substantial reduction in tumor size was reported using pegylated liposomes to enhance plasma half-life and tagging with folate (FA) for targeted distribution *in vivo (* 17). A hybrid nanoporous microparticle (hNP) carrier based on calcium carbonate and biopolymers derivatized with FA and carrying DOX as a chemotherapeutic drug model was created and evaluated on the human OS MG-63 cell line, which demonstrated reduced cell viability (76) (**Table 3** and **Figure 3**).

### Bisphosphonates as Targeting Ligands

Hydroxyapatite is a mineral needed for bone formation. BP have a high affinity for the hydroxyapatite matrix of bone because they chelate with the divalent calcium ions (Ca^2+^) in it (15, 113, 114). BP may be a promising targeted drug for treating bone cancer since it accumulates in bone and helps to limit osteoclast recruitment and adherence to the bone matrix, which diminishes osteoclast half-life and directly suppresses its activity.

Multifunctional alendronate-drug conjugates delivery systems are an emerging notion for successful OS targeted therapy. Morton et al. (77) demonstrated that alendronate-coated nanoparticles bind and internalize fast in human OS 143B cells. Pull-(GGPNle—PTX) was created by covalently conjugating PTX with pullulan and alendronate. This pullulan-alendronate-coated medication delivery method significantly reduced breast cancer growth, migration, and angiogenesis, as well as OS bone metastases (78). Zhao et al. created a new PTX NP coated with polydopamine and grafted with alendronate as a ligand for OS targeted therapy. *In vitro* experiments demonstrated that targeting NPs were more hazardous to K7M2 WT OS cells than non-targeting NPs (79). In another study, alendronate (ALN) was conjugated with hyaluronic acid and DSPEPEG2000COOH *via* a bioreducible disulfide linker (SS) to produce an ALNHASSL loaded with DOX. *In vitro*, ALNHASSLL/DOX shown increased cytotoxicity to human OS MG-63 cells, as well as high and quick cellular uptake; *in vivo*, ALNHASSLL/DOX demonstrated excellent tumor growth suppression and prolonged survival time for orthotopic OS nude mouse models. This work showed that ALNHASSLL/DOX, which has bone-and CD44-dual-targeting properties as well as redox sensitivity, could be a potential OS-targeted treatment (14).

Several BP-conjugated polymeric nanocarriers were created to carry chemotherapeutic medicines to OS, and their tumor-targeting and anticancer activities were tested *in vivo*. With the exception of free DOX or nontargeted DOX nanocarriers, these functionalized, DOX-loaded nanoparticles showed increased, longer tumor accumulation and dramatically better anticancer effectiveness (77, 82). Katrin et al. (80) created a DOX BP prodrug to target bone metastases. In human plasma, the prodrug exhibits rapid DOX release and appropriate stability over many hours. For about the first time, researchers found that using DOX-conjugated BP NPs reduced tumor growth 40% more effectively than using free DOX in a xenograft mouse model of human Saos-2 OS (82). It has been widely employed in the detection of bone formation and remodeling diseases, including bone cancers, using Methylene diphosphonate (MDP), a significant radiopharmaceutical agent (115). Wu et al. (81) created a phospholipid liposome coupled with MDP for *in vivo* targeting of OS and single photon emission computed tomography trace. By targeting OS, this method reduces toxicity to normal tissue while increasing cancer uptake (116). Recently, hydroxyapatite NPs functionalized with medronate (the smallest BP) as a bone-targeting moiety in OS focused treatment have been reported. *In vitro* studies revealed that JQ1-loaded hydroxyapatite NPs effectively suppressed OS cell migration and invasion while being less hazardous to primary fibroblasts (15). Bone-targeted rather than OS targeted, BP may have the capacity to suppress osteoclasts and bone homeostasis throughout their extended stay in bone tissue (113, 117).

OS tumors are more likely to accumulate DOX-loaded NPs when a combination of pamidronate and NP EPR is used to target bone (83). Yin et al. (84) demonstrated the use of pamidronate-functionalized nanoparticles to transport DOX to the bone microenvironment for the targeted treatment of OS. (**Table 3** and **Figure 3**).

## Conclusion

OS treatment is hindered by its unknown origin, high genetic instability, large histological heterogeneity, lack of diagnostic biomarkers, high local aggressiveness, and potential for rapid spread. Certain negative effects, including tissue damage, medication resistance, and rapid blood clearance are associated with chemical treatments for OS. It is possible to improve the capacity of medications to target cancer by using active targeting strategies, such as ligand-mediated tumor targeting. An eligible ligand is critical in this progression. We reviewed commonly utilized ligands as well as other compounds in this review; these conjugates are used as emerging tools for the treatment of OS. Although significant advances in the creation of new multifunctional ligand-chemical platforms may hold enormous promise for the treatment of OS in the future, these conjugates are not yet well-developed for usage in OS patients. The majority of them are still in the cellular and animal experimental stages, and there is a lengthy transition period before they can be used in humans. However, due to improved therapeutic effects and reduced side effects, as well as the growing use of next-generation sequencing and emerging technologies such as single-cell sequencing, which has resulted in the discovery of a large number of OS heterogeneity and novel targets, the active targeting strategy of ligand-based chemicals is doomed to play an important role in the treatment of OS.

## Future Perspectives

Beyond targeted ligand-chemodrug conjugates, surgery is a significant component in the treatment of OS. OS resection is tough due to the varied placement of tumors and its closeness with adjacent tissues. It also carries a considerable risk of postoperative complications. According to Ma et al., to overcome the difficulty of precise OS resection, computer-aided design was employed to create patient-specific guidance templates for OS resection based on CT scans and magnetic resonance imaging of human OS. The guiding templates were then created using a 3D printing technology. The OS surgery was directed by the guiding templates, which occurred in more exact removal of the tumorous bone and placement of the bone implants, less blood loss, a shorter operation duration, and less radiation exposure throughout the procedure. Patients recovered sufficiently enough to achieve a mean Musculoskeletal Tumor Society score, according to follow-up investigations (118). Bone grafts, which can be autogenous (from the own body of patient), homogeneous (from other individuals), or xenografts (from other species), are currently used to replace bone after surgery. Because each of these techniques has its own set of limitations, research has concentrated on the use of synthetic grafts that are both safer and more cost-effective (119). To be acceptable for bone regeneration, synthetic osteo-regenerative scaffolds must be biocompatible and have the requisite porosity, degradability, compositional, and mechanical qualities (120). The above conditions are achieved by 3D printing. 3D printed scaffolds can be precisely designed to mimic bone tissue morphologically (120) and provide control over scaffold pore shape and size (121), as well as facilitate the incorporation of other functional agents within the scaffold, making them an advantageous method for fabricating implantable scaffolds for bone regeneration (120). According to Jing et al., cisplatin/hydrogel-loaded 3D-printed titanium alloy implants are safe and effective for treating OS-related bone defects and should be explored for clinical application.

However, treating bone abnormalities caused by surgical resection may not be enough. There is still a chance that some tumor cells are left over, which could lead to OS recurrence. To address this, Fu et al. 3D printed a bioceramic free carbon-embedding larnite (larnite/C). The free carbon was added to aid a photothermal effect when an NIR laser was used to excite it. *In vivo*, the scaffold was able to kill human OS cells, slow tumor growth in naked mice, and encourage new bone production. *In vitro*, expression of rat bone mesenchymal stem cells may be aided by the scaffolds (122). Scaffolds made of different carbon sources, such as graphene oxide, may also have photothermal conversion characteristics. Ma et al. created graphene oxide (GO)-modified-tricalcium phosphate (GO-TCP) composite scaffolds with photothermal properties. *In vitro*, photothermal impacts caused considerable MG-63 OS cell death and reduced tumor growth in mice. Furthermore, as compared to plain-TCP scaffolds, the GO-TCP scaffolds showed superior osteogenic differentiation and new bone production (123).

Drug-loaded implants currently combine active pharmaceuticals with a biocompatible carrier and slowly release the drug after being implanted, allowing for local treatment (124). As a result, high drug concentrations at the location of interest are achieved while systemic drug exposure is reduced, avoiding undesired side effects in OS treatment (125). In this circumstance, the high degree of flexibility and controllability of 3D printing technique allows for the creation of complex forms with individualized dosages with variable release profiles, which improves local treatment. Fahimipour et al. recently developed a 3D printed gelatin/alginate/-TCP scaffold. The scaffold was subsequently coated with poly (D,L-lactic-co-glycolic acid), which encapsulated VEGF for long-term release (126). Wang et al. also created a unique technique based on 3D printed poly L-lactic acid drug carriers that has the ability to realize the potential of tailored local chemotherapy in the treatment of OS and could serve as a universal platform for anti-OS therapy (124).

**Figure 4:**
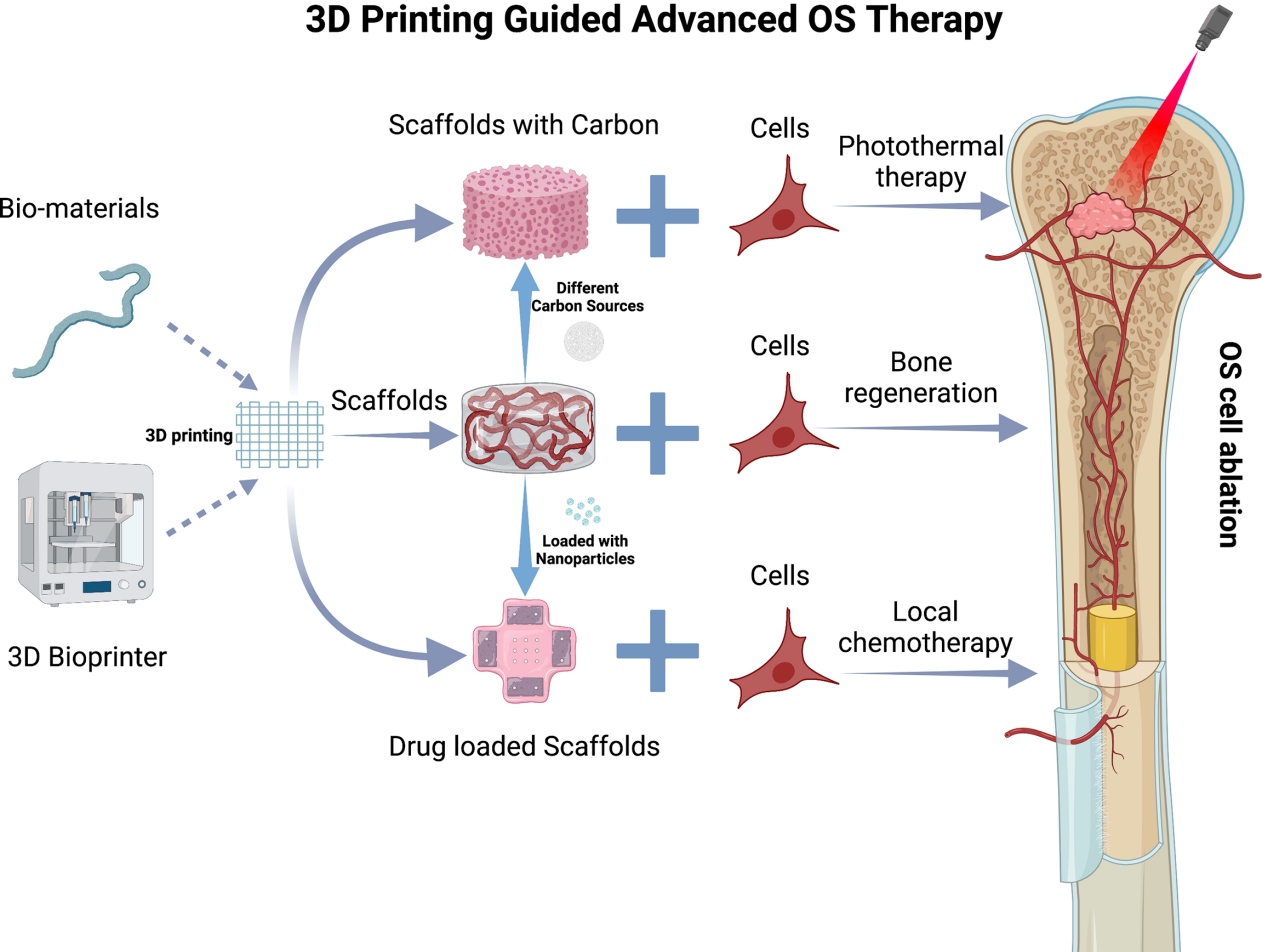
3D Printing guided advanced OS therapy. 3D printing scaffold and various types of carbon sources or nanocariers that may be incorporated. The nanocarriers and carbon sources are included in a 3D printed scaffold. The scaffold is then implanted into the critical defect site in the tibial due to OS resection to present bone regeneration, photothermal therapy, and local chemo release. This figure was created with BioRender.com.

Among these emerging insights to facilitate OS therapies, it should be noted that 3D printed scaffolds pose an opportunity with high potential. They may be used to provide templates in order to achieve precise OS resection, to enhance bone regeneration, to target residual OS cells after surgical resection, and to induce sustained release platforms for drugs (**Figure 4**).

## Author Contributions

CL and AL supervised and revised the manuscript. DX and ZW wrote and edited the manuscript. DG and JL provided the professional expertise. All authors contributed to the article and approved the submitted version.

## Funding

This review is supported by the National Natural Science Foundation Council of China (82172386 and 81922081 to CL), the Croucher Foundation (CAS14BU/CAS14201 to A.L.), the Department of Education of Guangdong Province (2021KTSCX104 to CL), the 2020 Guangdong Provincial Science and Technology Innovation Strategy Special Fund (Guangdong-Hong Kong-Macau Joint Lab) (2020B1212030006 to AL), the Guangdong Basic and Applied Basic Research Foundation (2020A1515011450 to JL), the Shenzhen Project of Science and Technology (JCYJ20190809094007719 to JL), the National Natural Science Foundation of China (82104216 to JL) and the Science, Technology and Innovation Commission of Shenzhen (JCYJ20210324104201005 to CL).

## Conflict of Interest

The authors declare that the research was conducted in the absence of any commercial or financial relationships that could be construed as a potential conflict of interest.

## Publisher’s Note

All claims expressed in this article are solely those of the authors and do not necessarily represent those of their affiliated organizations, or those of the publisher, the editors and the reviewers. Any product that may be evaluated in this article, or claim that may be made by its manufacturer, is not guaranteed or endorsed by the publisher.
